# Rethinking Cognitive Bias in Human–AI Cognitive Systems: A Structured Conceptual Mapping Review

**DOI:** 10.3390/bs16101829

**Published:** 2026-10-06

**Authors:** Alessandra Cecilia Jacomuzzi, Brigitta Pia Alioto, Sara Magni

**Affiliations:** Department of Philosophy and Cultural Heritage, Ca’ Foscari University of Venice, Calle Contarini 3484/D, Dorsoduro, 30123 Venice, Italy

**Keywords:** human–AI interaction, cognitive bias, automation bias, decision making, conceptual mapping review

## Abstract

Research in cognitive psychology has long shown that human judgment and decision making are systematically influenced by cognitive biases, particularly under conditions of uncertainty. As artificial intelligence (AI) systems become increasingly embedded in socially relevant decision making contexts—such as healthcare, education, communication, and governance—these cognitive dynamics do not disappear. Instead, they may be reproduced, amplified, or transformed through human–AI interaction. Despite the rapid growth of research on cognitive biases in AI-related contexts, existing reviews remain fragmented and heterogeneous, often focusing on single biases or specific application domains. This article reports a structured conceptual mapping review of review-level publications examining how cognitive biases are conceptualized, categorized, and addressed in human–AI interaction. A bounded search of Scopus, Web of Science, and PubMed identified ten eligible review publications published between 2020 and 2025. Using a three-level framework, the mapping distinguishes data-level, algorithmic-level, and human-interaction-level processes. Within the retrieved corpus, the analysis reveals heterogeneous terminology, a predominance of healthcare applications and interaction-level analyses, and limited review-level synthesis of empirical evaluations of mitigation strategies. Drawing on distributed cognition and joint cognitive systems, we propose coupled human–AI cognition as a theoretical lens rather than as an empirically established conclusion. The study provides a structured conceptual map, identifies terminological and methodological gaps, and develops propositions for future empirical research.

## 1. Introduction

A substantial body of research in cognitive psychology has demonstrated that human judgment and decision making may be shaped by systematic tendencies under uncertainty (Tversky & Kahneman, 1974). Kahneman’s dual-process theory further distinguishes between fast, intuitive processes (System 1) and slower, deliberative reasoning (System 2), identifying the former as a primary source of many cognitive biases (Kahneman, 2011). From a psychological perspective, these biases do not merely reflect individual errors, but rather systematic mechanisms that shape human interaction with complex decision environments and decision-support systems. Converging evidence from cognitive neuroscience suggests that these automatic processes are supported by neural circuits that can influence behavior before deliberative processing, making them relatively resistant to voluntary correction (LeDoux, 2000). These mechanisms affect a wide range of activities, including risk assessment, forecasting, and complex problem-solving.

At the same time, artificial intelligence (AI) systems are increasingly used to support or automate decision making in domains with significant social implications, such as healthcare, recruitment, education, governance, and digital communication. In many of these contexts, AI-based tools are introduced with the expectation of improving efficiency, consistency, and objectivity, often being portrayed as a corrective to human cognitive limitations (Logg et al., 2019; Jacomuzzi & Alioto, 2024). This narrative has contributed to the widespread assumption that algorithmic systems are inherently neutral or more rational than human decision-makers.

However, interdisciplinary scholarship has repeatedly challenged this assumption. Research in ethics, law, and science and technology studies has emphasized that AI systems are socio-technical artifacts shaped by human design choices, training data, and institutional contexts (Barocas & Selbst, 2016; Mittelstadt et al., 2016). As a result, algorithms may reproduce existing biases, introduce new forms of distortion, or influence human judgment through their interactional dynamics rather than eliminating bias altogether.

Within this broader debate, increasing attention has been devoted to the psychological mechanisms underlying human–AI interaction. Early work on automation showed that automated systems can alter users’ trust, responsibility attribution, and verification behavior, sometimes leading to misuse, disuse, or overreliance (Parasuraman & Riley, 1997). More recent studies have examined trust calibration, decision delegation, and perceived agency (Glikson & Woolley, 2020; Rastogi et al., 2020). The present study uses distributed cognition (Hutchins, 1995), joint cognitive systems (Hollnagel & Woods, 2005), and the extended-mind account of coupled cognition (Clark & Chalmers, 1998) as a sensitizing theoretical framework. On this view, decision making may be distributed across people, representations, artifacts, organizational practices, and environmental constraints. The framework does not presuppose that every bias is an emergent system property; rather, it directs attention to relations among human cognitive processes, algorithmic outputs, data representations, interface characteristics, and decision contexts.

Despite the rapid expansion of the literature, research on cognitive biases in human–AI interaction remains fragmented. Contributions originate from different disciplines, adopt heterogeneous terminologies, and focus on different levels of analysis. Importantly, this fragmentation is not only disciplinary but also conceptual: similar cognitive phenomena are often described using different labels, levels of explanation, or implicit psychological assumptions. As a consequence, the notion of “cognitive bias” risks functioning as an umbrella term lacking theoretical precision, particularly with respect to the underlying assumptions about human–AI cognitive systems. Reviews published to date tend to address specific biases or narrowly defined application domains, limiting opportunities for cross-domain comparison and theoretical integration.

When a field is characterized by heterogeneous terminology, disciplinary fragmentation, and conceptually overlapping constructs, conceptual mapping can clarify how central terms are defined, differentiated, and related (Grant & Booth, 2009; Snyder, 2019; Schreiber & Cramer, 2024). Unlike an umbrella review, which conventionally synthesizes systematic reviews and meta-analyses, a conceptual mapping review does not aggregate effect estimates or establish an evidence hierarchy. Its purpose is to construct a transparent representation of a conceptual landscape, identify ambiguities, and support theory development (Jaakkola, 2020; Schreiber & Cramer, 2024).

Accordingly, the present study conducts a structured conceptual mapping review of review-level publications addressing cognitive bias in human–AI interaction. The study maps application domains, levels and types of bias, roles attributed to AI, theoretical frameworks, cognitive mechanisms, psychological outcomes, and mitigation strategies. It also uses the framework proposed by Adepoju and Adepoju (2025) to distinguish data-level, algorithmic-level, and human-interaction-level processes. The resulting map is intended to identify patterns of conceptual attention within the retrieved corpus and to support a theoretically grounded account of human–AI cognitive systems, rather than to provide an exhaustive or definitive synthesis of empirical effects. For terminological clarity, in this work a heuristic is understood as a strategy that reduces cognitive complexity and is not necessarily erroneous or harmful. A cognitive bias refers more specifically to a systematic tendency that produces deviation relative to an explicitly or implicitly adopted standard of judgment. Data and algorithmic biases concern systematic distortions associated with datasets, model development, or system operation, whereas discriminatory or systemic bias refers to patterns that produce unjustified disadvantages for individuals or social groups (Barocas & Selbst, 2016; Mittelstadt et al., 2016). These categories may interact but should not be treated as conceptually interchangeable.

## 2. Research Questions

This structured conceptual mapping review addresses the following research questions (RQs):

- RQ1. How has the scientific literature conceptualized the interaction between humans and artificial intelligence across different application domains?
- RQ2. Which types and levels of bias are explicitly discussed in relation to human–AI interaction?
- RQ3. What roles of AI are described across domains, and how are they framed in relation to human decision making?
- RQ4. Which cognitive mechanisms and psychological outcomes are reported concerning human interaction with AI systems?
- RQ5. Which mitigation strategies are proposed in the review-level literature to address cognitive biases in human–AI interaction?

The research questions guided a structured mapping of the retrieved corpus; individual publications were not required to address all research questions simultaneously. Review counts are interpreted as patterns of conceptual attention, not as independent estimates of prevalence or evidential strength.

## 3. Method

### 3.1. Procedure

This study employed a structured conceptual mapping review design to examine how cognitive bias is conceptualized across review-level publications on human–AI interaction (Grant & Booth, 2009; Snyder, 2019; Schreiber & Cramer, 2024). This design was selected because the objective was not to aggregate effect estimates or establish an evidence hierarchy, but to identify terminology, conceptual categories, levels of analysis, proposed mechanisms, reported outcomes, and mitigation strategies across the heterogeneous secondary literature. Relevant Preferred Reporting Items for Systematic Reviews and Meta-Analyses (PRISMA) 2020 items were used to report search and selection procedures transparently; PRISMA was used as a reporting framework and not as a designation of the review methodology (Page et al., 2021).

The unit of analysis was the review-level publication. Eligible publications included narrative, conceptual, literature, systematic, scoping, and interdisciplinary reviews that explicitly addressed cognitive bias or cognitive processes in relation to artificial intelligence systems or human–AI interaction. Primary empirical studies, technical reports, and publications focusing exclusively on statistical or fairness-related bias without reference to human cognitive processes or interaction were excluded. The inclusion of heterogeneous review types was intentional because the aim concerned conceptualization rather than effect estimation.

A structured, bounded literature search was conducted in September 2025 across Scopus, Web of Science, and PubMed. These databases were selected to provide coverage across psychology, interdisciplinary social science, and biomedical research. The search was not designed to provide exhaustive coverage of the computer science and human–computer interaction (HCI) conference literature; the omission of ACM Digital Library and IEEE Xplore is therefore treated as a limitation of corpus coverage.

**Figure 1:**
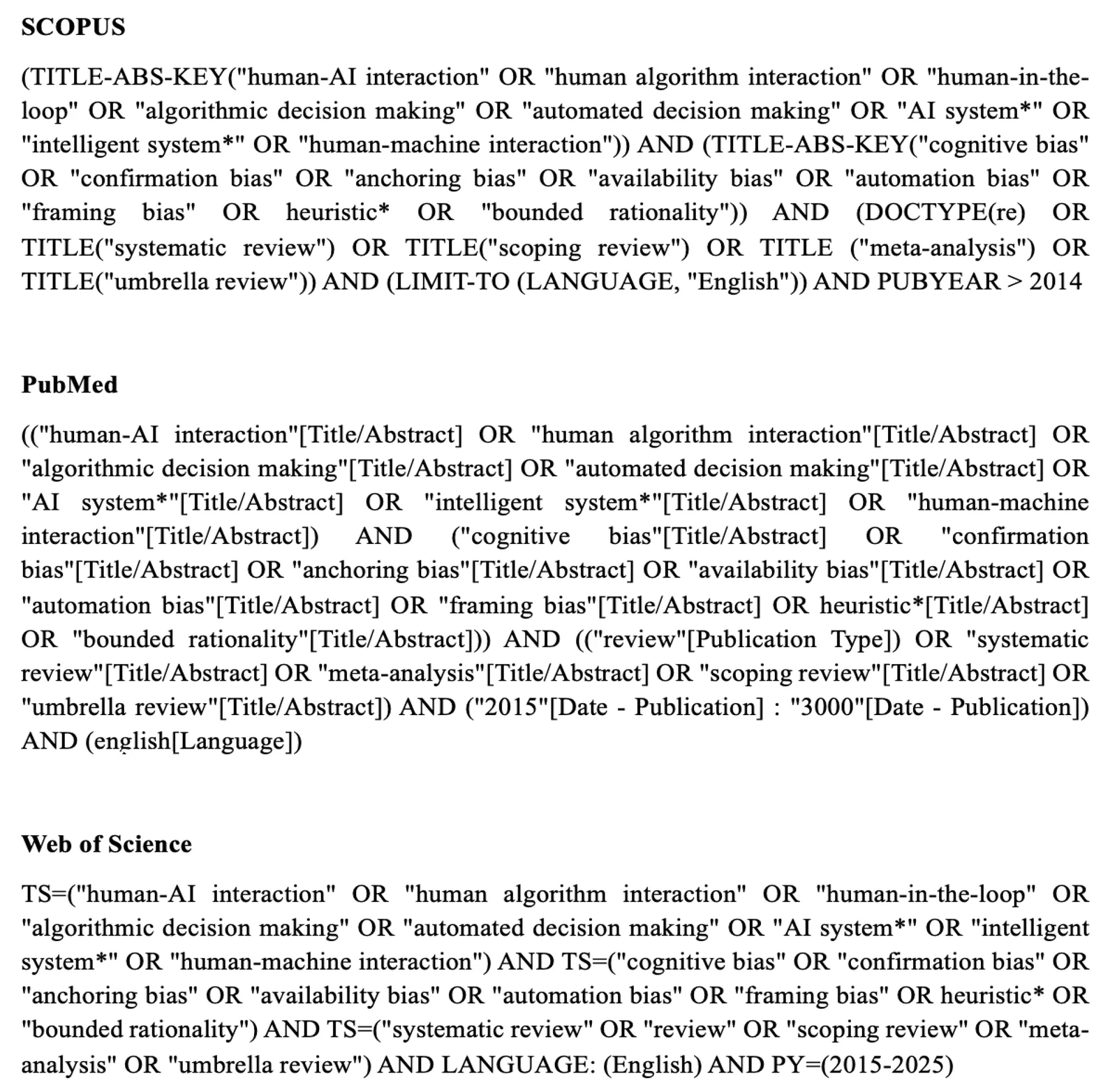
Search strings on Scopus, PubMed and Web of Science. The asterisk (*) denotes the truncation wildcard.

The search combined terms related to artificial intelligence, cognitive bias, and review-level publications. Search strings were adapted to the syntax of each database and are reported in Figure 1. Because the search strategy was intentionally retained as originally executed, findings are restricted to the retrieved corpus and should not be generalized to all the primary or secondary literature on human–AI interaction.

The search was restricted to publications written in English and published between 2015 and 2025. This time frame was selected to capture the period in which contemporary AI systems and human–AI interaction became prominent research topics.

After duplicate removal, title and abstract screening was conducted independently by two researchers using the predefined eligibility criteria (Table 1). One researcher additionally repeated the initial assessment as an internal consistency check. Potentially eligible publications were subsequently assessed at full text independently by both researchers. Following each stage, the researchers compared their decisions, and discrepancies were resolved through discussion and consensus.

### 3.2. Data Extraction and Conceptual Mapping

**Figure 2:**
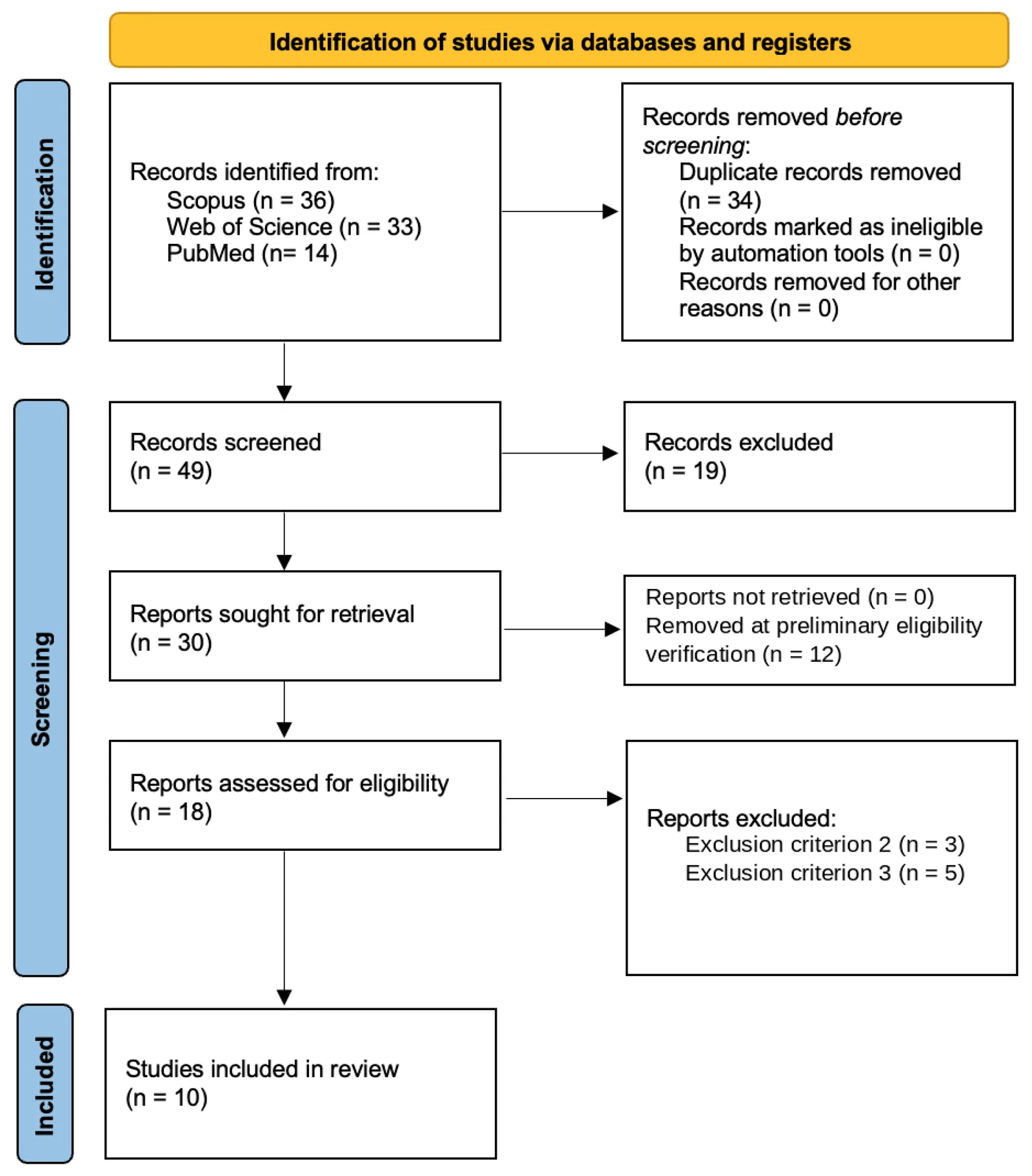
PRISMA 2020 flow diagram.

The three database outputs were merged and duplicates were removed, yielding 49 records for title and abstract screening. Nineteen records were excluded at this stage. Of the 30 records retained after initial screening, 12 were removed during preliminary retrieval and eligibility verification, leaving 18 publications for formal full-text assessment. Eight of the 18 full texts were excluded under eligibility criteria 2 or 3, and ten publications were included in the final conceptual map (Figure 2). The category-specific reasons for the intermediate removal of the 12 records were not preserved in the archived pre-consensus screening matrices and cannot be reconstructed retrospectively; this reporting limitation is acknowledged below.

The two researchers independently extracted and coded all included publications using a structured data-extraction form aligned with the five research questions. Coding covered publication characteristics, application domains, levels and types of bias, roles attributed to AI, theoretical frameworks, cognitive mechanisms, psychological outcomes, and mitigation strategies. The researchers compared their coding matrices item by item and resolved differences through discussion, reference to the full text, and consensus. A formal inter-rater agreement coefficient could not be calculated because the original pre-consensus screening and coding matrices were not retained.

First-order coding preserved constructs explicitly named or clearly reported in the included publications, minimizing interpretative overreach. A second-order conceptual mapping stage then compared potentially overlapping constructs and organized them into broader analytical categories. Original source labels and author-generated interpretative categories were kept analytically distinct. This two-stage strategy balances fidelity to source terminology with the need to address jingle-jangle problems, in which identical labels may denote different constructs and different labels may denote similar phenomena. 

## 4. Results

All results derive exclusively from the ten review-level publications included in the structured conceptual mapping review. Counts indicate the number of publications in which a construct was identified and should not be interpreted as independent confirmations of prevalence, causal effects, or evidential strength.

### 4.1. General Overview

#### 4.1.1. Temporal Distribution

The publications included in the conceptual map were published between 2020 and September 2025: one in 2020, one in 2021, two in 2022, none in 2023, two in 2024, and four in the partial 2025 search year.

**Figure 3:**
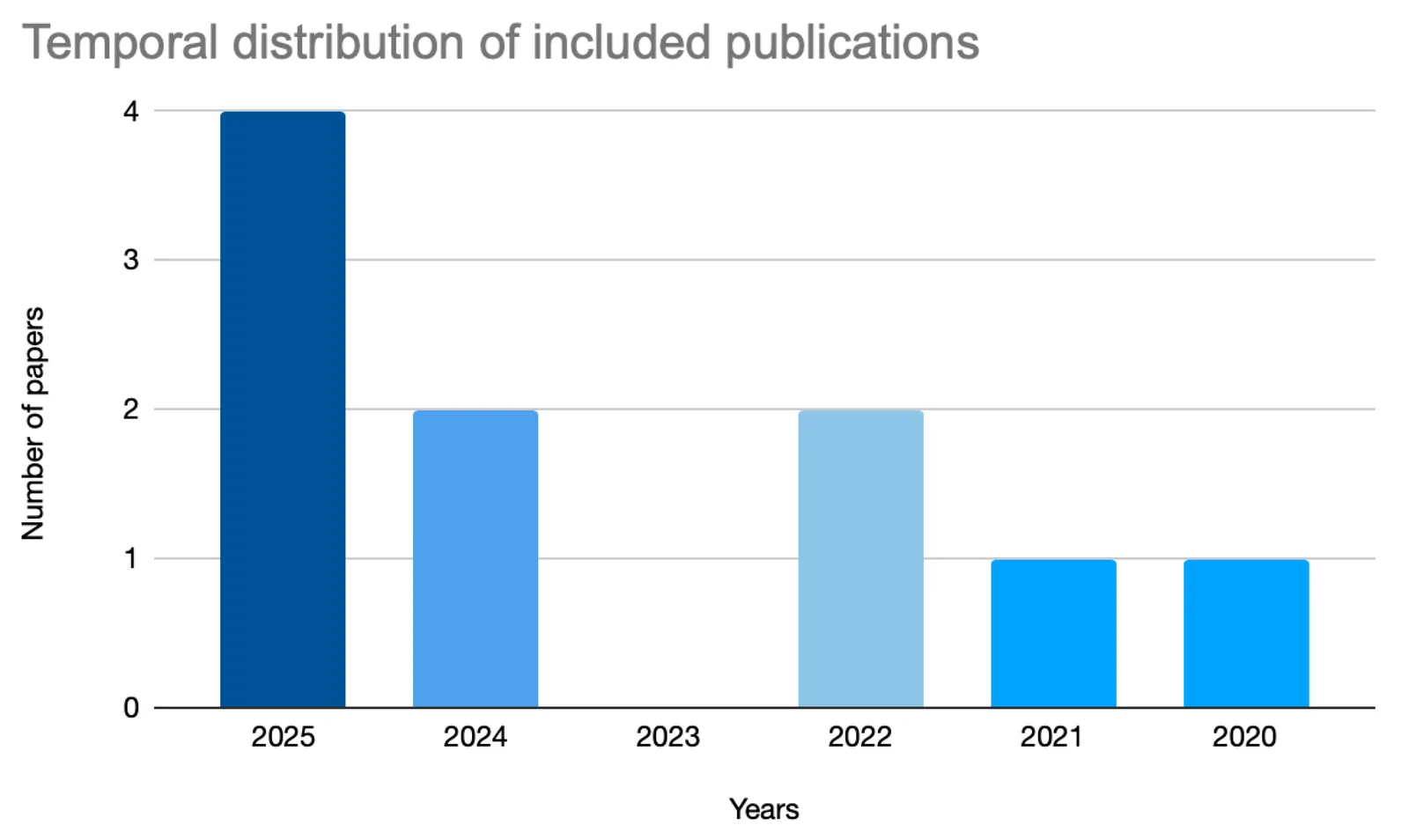
Descriptive temporal distribution of included publications (2023 = 0; 2025 coverage through September).

Given the small corpus and incomplete coverage of 2025, this distribution is reported descriptively and should not be interpreted as evidence of a temporal trend or recent intensification (Figure 3).

#### 4.1.2. Type of Review

All included publications were classified as non-systematic reviews. Review types were mapped using distinctions described by Grant and Booth (2009), Paré et al. (2015), and Snyder (2019) and are reported in Table 2.

None of the included studies self-identifies as a systematic review or meta-analysis. Overall, the corpus is characterized by exploratory, interpretative, and conceptual approaches, rather than by quantitative synthesis of evidence.

#### 4.1.3. Stated Purpose of the Reviews

Based on the stated aims of the included reviews, four main purpose categories were identified in Table 3.

Across all reviews, the primary focus is not on technical metrics, but rather on how AI systems are interpreted, trusted, and used by humans, as well as on the cognitive, ethical, and organizational implications of such use (Table 4).

### 4.2. RQ1. How Has the Scientific Literature Conceptualized the Interaction Between Humans and Artificial Intelligence Across Different Application Domains?

With respect to the first research question, the dataset indicates that human–AI interaction is conceptualized in a context-dependent and domain-specific manner across the included reviews while consistently being framed as more than a purely technical interface.

**Figure 4:**
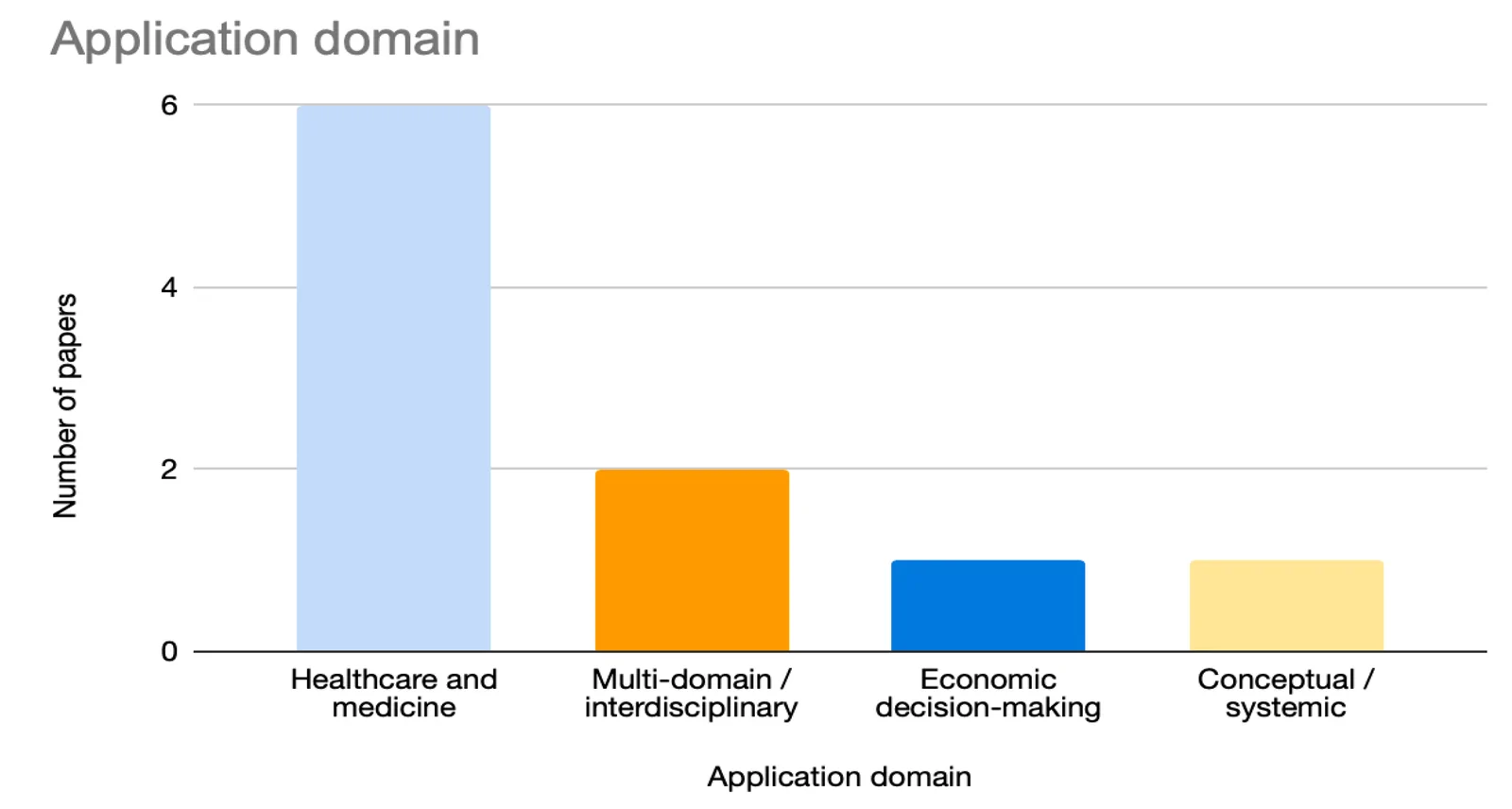
Application domains represented in the retrieved corpus.

As shown in Figure 4, six of the ten included reviews conceptualized human–AI interaction within healthcare-related domains, including radiology, gastrointestinal endoscopy, dentistry, and clinical decision-support systems. This concentration is a characteristic of the retrieved corpus and not an estimate of the domain distribution of the wider field.

Two reviews adopted a multi-domain and interdisciplinary perspective on human–AI interaction (Korteling et al., 2021; Chugunova & Sele, 2022). Korteling et al. (2021) described interaction as a form of collaboration between humans and intelligent systems, with an emphasis on task allocation and users’ understanding of AI capabilities. Chugunova and Sele (2022), drawing on experimental evidence, conceptualize interaction with AI agents as qualitatively different from human–human interaction, particularly in terms of emotional and social engagement.

Only one review conceptualized human–AI interaction explicitly within the domain of economic decision making (Rodriguez-Fernandez, 2025). In this case, interaction was described as a cognitive environment shaping decision processes over time, rather than as a neutral support tool.

Finally, one review (Arora, 2020) addressed human–AI interaction at a conceptual and systemic level, discussing AI as a transformative element in professional practice without anchoring the analysis to a specific application domain. 

Across the included publications, human–AI interaction was most often conceptualized as a process embedded in professional, organizational, or social contexts rather than as a purely technical exchange (Table 5).

### 4.3. RQ2. Which Types and Levels of Bias Are Explicitly Discussed in Relation to Human–AI Interaction?

The second research question concerns the types and levels of bias explicitly analyzed in the included publications (Table 6). Human and interaction-level bias was the most frequently coded level within the retrieved corpus; this descriptive concentration does not establish the prevalence of such bias in human–AI systems generally.

Six reviews explicitly describe human and interaction-level bias, focusing on how users’ cognitive processes influence interaction with AI systems (Padhani & Papanikolaou, 2025; Aldosari et al., 2025; Korteling et al., 2021; Campion et al., 2024; Tuygunov et al., 2025; Chugunova & Sele, 2022). In these reviews, bias was discussed in relation to reliance on AI outputs, trust calibration, decision delegation, and reduced critical oversight during interaction.

Algorithmic-level bias was explicitly analyzed in three reviews (Koçak et al., 2024; Tuygunov et al., 2025; Rodriguez-Fernandez, 2025). In these reviews, bias was linked to model design, training procedures, or system-level characteristics, and was typically discussed in terms of its downstream effects on human decision making rather than as an isolated technical issue.

Data-level bias was explicitly addressed in only one review (Koçak et al., 2024), in which unrepresentative and historically biased datasets were discussed as a foundational source of downstream bias in medical imaging AI.

Two reviews (Arora, 2020; Knop et al., 2022) did not explicitly classify bias according to level and were therefore coded as not reported (NR), although they discussed risks, challenges, and limitations associated with AI systems more broadly.

First-order results included only bias labels explicitly named in the source publications, such as automation bias, algorithmic bias, data bias, anchoring bias, confirmation bias, framing effects, algorithm aversion, alarm fatigue, and deskilling. Behaviors not labeled as biases were retained using the source terminology. During second-order mapping, framework-level concepts, cognitive processes, interactional behaviors, proximal outcomes, and longitudinal capability changes were distinguished to avoid treating them as equivalent constructs.

### 4.4. RQ3. What Roles of AI Are Described Across Domains, and How Are They Framed in Relation to Human Decision Making?

The third research question addresses the roles attributed to AI systems across domains and how these roles are framed in relation to human decision making (Table 7).

Across the included reviews, AI was most frequently described as a support tool, a role reported in seven reviews (Padhani & Papanikolaou, 2025; Aldosari et al., 2025; Koçak et al., 2024; Campion et al., 2024; Tuygunov et al., 2025; Knop et al., 2022; Arora, 2020). In these reviews, AI systems were described as assisting human decision-makers by providing additional information, risk assessments, or diagnostic suggestions, while final responsibility was reported to remain with the human user.

AI was explicitly discussed as a recommendation system in two reviews (Padhani & Papanikolaou, 2025; Rodriguez-Fernandez, 2025). In clinical contexts, recommendations were described as inputs requiring human validation, whereas in the economic domain they were discussed in terms of their influence on preferences and choices.

Automation was explicitly addressed in four reviews (Aldosari et al., 2025; Campion et al., 2024; Korteling et al., 2021; Arora, 2020). In these reviews, automation was predominantly discussed as a risk or challenge rather than as a desired end state. Concerns associated with automation included over-reliance, reduced vigilance, loss of skills, and diminished human agency.

None of the included reviews described scenarios involving the full replacement of human decision-makers or the complete delegation of decision authority to AI systems.

### 4.5. RQ4. Which Cognitive Mechanisms and Psychological Outcomes Are Reported Concerning Human Interaction with AI Systems?

Across the ten included publications, reported constructs were heterogeneous and unevenly distributed. To address differences in level of analysis, bounded rationality was treated as a theoretical framework; heuristic processing as a cognitive process; trust as a relational state requiring calibration; reliance, delegation, and verification as interactional behaviors; reduced vigilance and uncritical acceptance as proximal outcomes; and deskilling as a longitudinal capability outcome. The original labels remain visible in Table 8, while these distinctions guide the interpretation.

The source publications used over-reliance both as a proposed mechanism and as an outcome. Rather than assuming equivalence, the conceptual map treats reliance as an interactional behavior whose antecedents and consequences depend on context. Similarly, reduced critical oversight, reduced vigilance, and uncritical acceptance are treated as related but non-identical constructs involving verification behavior, monitoring, and decision acceptance, respectively.

From the standpoint of psychological outcomes, the dataset shows an equally dispersed pattern. Only one review reports reduced vigilance as an outcome (Padhani & Papanikolaou, 2025), while uncritical acceptance of AI recommendations is reported explicitly only in Aldosari et al. (2025). A further two reviews describe outcomes that are best captured as “concerns”: Knop et al. (2022) report acceptance concerns and fear of biasing, and Tuygunov et al. (2025) report accountability and reliance concerns. Over-reliance also appears as an outcome in two reviews (Campion et al., 2024; Chugunova & Sele, 2022), with Campion et al. (2024) additionally reporting deskilling. The interdisciplinary review by Chugunova and Sele (2022) is the only one reporting reduced emotional response, whereas the economic psychology review by Rodriguez-Fernandez (2025) is the only one reporting preference reshaping (together with “bias amplification”). Finally, Koçak et al. (2024) is the only review in which outcomes are coded as compromised clinical judgment and a false objectivity perception, and Korteling et al. (2021) uniquely reports misunderstanding AI capabilities as an outcome. None of the included reviews provides quantitative synthesis of psychological outcomes; rather, outcomes are presented narratively or conceptually, as reflected in the coding.

### 4.6. RQ5. Which Mitigation Strategies Are Proposed in the Review-Level Literature to Address Cognitive Biases in Human–AI Interaction?

Mitigation strategies in the included publications were generally presented as recommendations or design principles rather than as synthesized evaluations of intervention effectiveness (Table 9). This corpus-level finding should not be interpreted as evidence that empirical evaluations are absent from the primary literature; for example, Buçinca et al. (2021) experimentally evaluated cognitive forcing functions intended to reduce AI overreliance.

Human-in-the-loop approaches are proposed in two reviews (Padhani & Papanikolaou, 2025; Aldosari et al., 2025), which emphasize maintaining human oversight of AI outputs and final human responsibility for decisions. A different cluster of strategies concerns transparency and user understanding. Explainability is proposed in one review (Knop et al., 2022), while interpretability and related measures (e.g., clinician understanding and auditability) are proposed in one review (Arora, 2020). In other words, these strategies seem to be present, but not widespread across the corpus.

Strategies related to education and capability-building appear in four reviews: Korteling et al. (2021) propose education and intelligence awareness training; Campion et al. (2024) propose clinician training; Tuygunov et al. (2025) explicitly code AI literacy; and Rodriguez-Fernandez (2025) includes education among proposed measures. Governance-related strategies are present in three reviews (Aldosari et al., 2025; Tuygunov et al., 2025; Rodriguez-Fernandez, 2025), reflecting recurring concerns about ethical or systemic management of AI adoption.

Finally, strategies targeting upstream technical sources of bias are comparatively rare in the dataset. Database diversification and related measures are coded only in Koçak et al. (2024), which is also the only review explicitly coded at the data level. Human-centered design appears explicitly only once, in Campion et al. (2024). Moreover, continuous evaluation/monitoring (as coded) appears only in Arora (2020), where it is framed through auditability and related practices, whereas Chugunova and Sele (2022) report NR (Not Reported) for mitigation strategies. Taken together, these patterns indicate that mitigation strategies are not only largely normative, but also fragmented across the corpus, with different domains prioritizing different levers (oversight, explainability, education, governance, or data practices).

## 5. Discussion

The present structured conceptual mapping review examined how cognitive biases are conceptualized, categorized, and addressed across ten heterogeneous review-level publications. Its contribution is a bounded conceptual map of application domains, bias levels, AI roles, theoretical frameworks, cognitive processes, interactional behaviors, psychological outcomes, and mitigation strategies.

Within the retrieved corpus, terminology is conceptually rich but methodologically and theoretically uneven. The recurrence of interaction-level constructs indicates where these publications direct attention; it does not establish their empirical prevalence in the wider field. The two-stage coding strategy also shows that some apparent fragmentation arises from differences in labeling and level of explanation.

Human and interaction-level bias was the most frequently coded level in the corpus. The publications most often discussed trust calibration, reliance on system outputs, delegation of decision authority, verification behavior, and reduced critical oversight. This pattern is compatible with—but does not independently demonstrate—a coupled-systems interpretation of cognitive bias.

Drawing on distributed cognition (Hutchins, 1995), joint cognitive systems (Hollnagel & Woods, 2005), and coupled cognition (Clark & Chalmers, 1998), we propose that some systematic distortions may arise through relations among human cognition, algorithmic outputs, interfaces, data representations, organizational practices, and task constraints. Emergence is therefore advanced here as a theoretical proposition generated through conceptual mapping, not as an empirical conclusion established by the ten included publications.

This distinction matters because identical behavioral outcomes may be produced by different system configurations. Overreliance, for example, may reflect human heuristics, perceived algorithmic authority, interface presentation, organizational incentives, or combinations of these factors. Future studies should operationalize these components and test their dynamic relations, potentially through formal models of trust calibration, Bayesian updating, and bias amplification. Developing such a model is beyond the evidential scope of the present mapping review.

This emphasis is consistent with earlier work in the psychology of automation, which demonstrated that automated systems can systematically influence users’ monitoring behavior, trust, and responsibility attribution (Parasuraman & Riley, 1997). More recent research has similarly shown that AI systems shape human judgment not only through their outputs, but also through interactional dynamics and perceived authority (Glikson & Woolley, 2020; Rastogi et al., 2020).

The concentration of interaction-level discussion should not be interpreted as evidence that data-level or algorithmic bias is negligible. It indicates only that the retrieved review-level corpus concentrates more heavily on how humans interpret and use AI systems. The original search and review-level eligibility criteria may also have contributed to this distribution.

Six of the ten included publications addressed healthcare or clinical decision making. This concentration supports detailed consideration of professional responsibility, expertise, uncertainty, and safety-critical decision support, but restricts transferability to education, public administration, consumer-facing applications, conversational agents, and everyday recommender systems.

Cognitive phenomena such as deskilling, reduced vigilance, or compromised judgment may manifest differently in lower-stakes or non-professional settings. Consequently, the patterns identified in this review are likely to be more robust for professional decision-making contexts than for everyday or consumer-facing AI applications. At the same time, the limited number of review-level syntheses addressing domains such as education, public administration, or digital communication highlights an important gap in the literature and a priority for future reviews.

With respect to cognitive mechanisms, the findings point to a fragmented theoretical landscape. Although trust emerges as the most frequently reported mechanism, other processes—including heuristic processing, bounded rationality, mental models of AI, and emotional distancing—are discussed only sporadically across the reviewed literature. This fragmentation mirrors longstanding observations in cognitive psychology, where multiple theoretical traditions often coexist without systematic integration (Kahneman, 2011).

Similarly, psychological outcomes are reported almost exclusively in qualitative or narrative terms, and none of the included reviews provides a quantitative synthesis of psychological effects. This pattern is consistent with previous observations that research on human–AI interaction often prioritizes conceptual analysis and design implications over outcome measurement (Glikson & Woolley, 2020), thereby limiting cumulative knowledge at the psychological level.

Mitigation recommendations in the included publications were predominantly normative and principle-based. The reviews rarely synthesized empirical evaluations of mitigation effectiveness, but this should not be generalized to the entire primary literature. Empirical work, including Buçinca et al. (2021), shows that interaction-level interventions can reduce overreliance while also producing trade-offs in user experience and differential benefits. Future reviews should therefore distinguish the presence of a recommendation from evidence concerning its effectiveness, boundary conditions, and unintended consequences.

A further conceptual blind spot concerns the implicit construction of a “standard user.” Human–AI systems may disadvantage people whose cognitive characteristics, sensory capacities, age-related needs, or digital literacy do not correspond to assumed interaction patterns. From a coupled-systems perspective, such disadvantage may arise from a mismatch among user characteristics, interface demands, explanation formats, institutional expectations, and available accommodations. Because cognitive diversity and reasonable accommodation were not systematically represented in the retrieved corpus, they are identified here as theoretically derived research and human-centered-design priorities rather than direct review findings.

## 6. Methodological Considerations and Limitations

Several limitations should be acknowledged. First, the corpus is small, methodologically heterogeneous, and dominated by healthcare publications. Second, the bounded search covered Scopus, Web of Science, and PubMed but not ACM Digital Library, IEEE Xplore, or the broader HCI conference literature. Its terminology may also have under-retrieved publications using adjacent constructs such as human–AI teaming, algorithm appreciation, algorithm aversion, or overreliance. Findings therefore characterize the retrieved corpus rather than the complete literature. Third, overlap among primary sources cited by the included reviews was not formally quantified; review counts may therefore reflect repeated use of foundational studies and are interpreted only as patterns of conceptual attention. Fourth, no formal quality appraisal of the included narrative reviews was conducted, so conceptual contributions were mapped irrespective of methodological rigor.

Finally, the review-level design cannot determine the prevalence, causal effects, or effectiveness of cognitive biases and mitigation strategies in primary empirical studies.

## 7. Implications and Future Directions

This structured conceptual mapping review clarifies how cognitive bias is described across a bounded and heterogeneous corpus of review-level publications. It identifies recurrent constructs, terminological ambiguity, domain concentration, and gaps in the synthesis of mitigation evidence. Its principal theoretical contribution is a coupled-systems framework that organizes relations among human cognition, algorithmic processes, data, interfaces, and contexts while remaining a proposition for future empirical testing rather than a definitive meta-synthesis.

Future research should test the proposed system-level relations across clinical and non-clinical domains, distinguish theoretical frameworks from mechanisms and outcomes, evaluate mitigation strategies empirically, assess overlapping and discriminatory consequences, and incorporate cognitive diversity and reasonable accommodation into human-centered AI design.

## Figures and Tables

**Table 1:** Inclusion and exclusion criteria.

Inclusion Criteria	Exclusion Criteria
Reviews (e.g., narrative, systematic, scoping, umbrella reviews and meta-analyses).	Primary empirical studies and other non-peer-reviewed publications.
2. Reviews addressing artificial intelligence systems in relation to human decision making, judgment or interactions.	2. Studies not addressing artificial intelligence systems or algorithmic decision making.
3. Reviews that addressed cognitive bias, decision bias or cognitive processes in the context of AI systems or human–AI interaction.	3. Studies focusing exclusively on technical bias, statistical bias, or fairness in algorithms or datasets without reference to human cognitive processes or interactions.
4. Publications written in English.	4. Publications written in languages other than English.

**Table 2:** Type of review of the included studies.

Type of Review	Number of Reviews
Narrative/conceptual reviews	6
Literature reviews (non-systematic)	1
Mini-reviews/special reports	2
Interdisciplinary experimental review	1

**Table 3:** Stated purposes of the included reviews.

Stated Purpose Category	Reviews
Understanding human–AI interaction	Padhani and Papanikolaou (2025) Campion et al. (2024) Knop et al. (2022) Chugunova and Sele (2022)
Analyzing bias and risks of AI	Koçak et al. (2024) Aldosari et al. (2025) Tuygunov et al. (2025)
Conceptualizing AI as innovation or intelligence	Arora (2020) Korteling et al. (2021)
Exploring cognitive and psychological influence	Rodriguez-Fernandez (2025)

**Table 4:** Characteristics of the included review-level publications.

Review	Year	Mapped Review Type	Domain	Stated Focus
Padhani and Papanikolaou (2025)	2025	Narrative/conceptual	Healthcare	Human–AI interaction in prostate magnetic resonance imaging
Aldosari et al. (2025)	2025	Mini-review/special report	Healthcare	Risks and challenges of AI in medicine
Tuygunov et al. (2025)	2025	Mini-review/special report	Healthcare	Promise and risks of AI in dentistry
Rodriguez-Fernandez (2025)	2025	Narrative/conceptual	Economic decision making	Algorithmic cognitive influence
Koçak et al. (2024)	2024	Narrative/conceptual	Healthcare	Bias in medical-imaging AI
Campion et al. (2024)	2024	Narrative/conceptual	Healthcare	Human–AI interaction in endoscopy
Knop et al. (2022)	2022	Literature review (non-systematic)	Healthcare	Human factors in clinical decision support
Chugunova and Sele (2022)	2022	Interdisciplinary experimental review	Multi-domain	Experimental human–machine interaction
Korteling et al. (2021)	2021	Narrative/conceptual	Multi-domain	Human versus artificial intelligence
Arora (2020)	2020	Narrative/conceptual	Conceptual/systemic	AI as healthcare innovation

**Table 5:** Application domains and conceptualizations of human–AI interaction (RQ1).

Application Domain	Number of Reviews	Reviews (Author, Year)
Healthcare and medicine	6	Padhani and Papanikolaou (2025) Aldosari et al. (2025) Koçak et al. (2024) Campion et al. (2024) Tuygunov et al. (2025) Knop et al. (2022)
Multi-domain/interdisciplinary	2	Korteling et al. (2021) Chugunova and Sele (2022)
Economic decision making	1	Rodriguez-Fernandez (2025)
Conceptual/systemic (no specific domain)	1	Arora (2020)

**Table 6:** Levels of bias analyzed (RQ2).

Bias Level	Number of Reviews	Reviews (Author, Year)
Human and interaction-level bias	6	Padhani and Papanikolaou (2025) Aldosari et al. (2025) Korteling et al. (2021) Campion et al. (2024) Tuygunov et al. (2025) Chugunova and Sele (2022)
Algorithmic-level bias	3	Koçak et al. (2024) Tuygunov et al. (2025) Rodriguez-Fernandez (2025)
Data-level bias	1	Koçak et al. (2024)
Bias level not explicitly reported	2	Arora (2020) Knop et al. (2022)

**Table 7:** Roles of AI.

Role of AI	Number of Reviews	Reviews (Author, Year)
Support	7	Padhani and Papanikolaou (2025) Aldosari et al. (2025) Koçak et al. (2024) Campion et al. (2024) Tuygunov et al. (2025) Knop et al. (2022) Arora (2020)
Recommendation	2	Padhani and Papanikolaou (2025) Rodriguez-Fernandez (2025)
Automation (as risk/challenge)	4	Aldosari et al. (2025) Campion et al. (2024) Korteling et al. (2021) Arora (2020)

**Table 8:** Source labels for cognitive mechanisms and psychological outcomes (RQ4).

Category	Item	Number of Reviews	Reviews (Author, Year)
Cognitive mechanisms	Trust	5	Padhani and Papanikolaou (2025) Knop et al. (2022) Korteling et al. (2021) Campion et al. (2024) Chugunova and Sele (2022)
	Over-reliance	2	Padhani and Papanikolaou (2025) Aldosari et al. (2025)
	Reduced critical oversight	1	Aldosari et al. (2025)
	Bounded rationality	1	Rodriguez-Fernandez (2025)
	Heuristic processing	1	Rodriguez-Fernandez (2025)
	Mental models of AI	1	Korteling et al. (2021)
	Emotional distancing	1	Chugunova and Sele (2022)
Psychological outcomes	Reduced vigilance	1	Padhani and Papanikolaou (2025)
	Uncritical acceptance of AI recommendations	1	Aldosari et al. (2025)
	Over-dependence on AI	1	Arora (2020)
	Over-reliance	2	Campion et al. (2024) Chugunova and Sele (2022)
	Deskilling	1	Campion et al. (2024)
	Reduced emotional response	1	Chugunova and Sele (2022)
	Preference reshaping	1	Rodriguez-Fernandez (2025)
	Acceptance concerns; fear of biasing	1	Knop et al. (2022)
	Accountability and reliance concerns	1	Tuygunov et al. (2025)
	Compromised clinical judgment; false objectivity perception	1	Koçak et al. (2024)
	Misunderstanding AI capabilities	1	Korteling et al. (2021)

**Table 9:** Mitigation strategies reported in the included publications (RQ5).

Mitigation Strategy	Number of Reviews	Reviews (Author, Year)
Human-in-the-loop	2	Padhani and Papanikolaou (2025) Aldosari et al. (2025)
Explainability	1	Knop et al. (2022)
Interpretability/auditability/clinician understanding	1	Arora (2020)
Education/training/AI literacy	4	Korteling et al. (2021) Campion et al. (2024) Tuygunov et al. (2025) Rodriguez-Fernandez (2025)
Regulation/governance/ethical guidelines	3	Aldosari et al. (2025) Tuygunov et al. (2025) Rodriguez-Fernandez (2025)
Dataset diversification/bias detection/transparency/fairness-aware design	1	Koçak et al. (2024)
Human-centered design	1	Campion et al. (2024)
NR (no mitigation strategy coded)	1	Chugunova and Sele (2022)

## Data Availability

The data presented in this study are available within the article. Additional extraction data are available from the corresponding author upon reasonable request, as they consist of internal coding files that are not publicly archived.
